# Supplementary material for: Activating p53 abolishes self-renewal of quiescent leukaemic stem cells in residual CML disease
Source: Nat Commun. 2024 Jan 22;15:651. doi: 10.1038/s41467-024-44771-9 (PMC10800356; doi:10.1038/s41467-024-44771-9)
Supplement: Supplementary file 1 — Supplementary Information [file 41467_2024_44771_MOESM1_ESM.pdf]

SUPPLEMENTARY INFORMATION

Activating p53 abolishes self-renewal of quiescent leukaemic stem cells in residual CML disease

Mary T. Scott[1,*], Wei Liu[1,*], Rebecca Mitchell[2], Cassie J. Clarke[2], Ross Kinstrie[1], Felix Warren[2], Hassan Almasoudi[1,3], Thomas Stevens[1], Karen Dunn[2], John Pritchard[1], Mark E. Drotar[1,2], Alison M. Michie[2], Heather G. Jørgensen[2], Brian Higgins[4], Mhairi Copland[2] and David Vetrie[1]

*contributed equally to this study

[1] Wolfson Wohl Cancer Research Centre,
Institute of Cancer Sciences,
University of Glasgow,
Glasgow, United Kingdom

[2] Paul O'Gorman Leukaemia Research Centre,
Institute of Cancer Sciences,
University of Glasgow,
Glasgow, United Kingdom

[3]Department of Clinical Laboratory Sciences,
College of Applied Medical Sciences,
Najran University, Najran, Saudi Arabia

[4] Genentech Inc,
South San Francisco, CA
USA

Supplementary Table 1. Primary $CD34^+$ cell samples used in this study.

Sample IDs	Experiment	Age Ranges of Individuals	Sexes of individuals	CML disease stages
CML I-VI	in vitro drug treatments	20-63	2 male, 4 female	Chronic or blast phase CML
CML VII-IX	RNA-seq of drug treated patient samples $CD34^+$ cells	27-57	1 male, 2 female	Chronic phase CML
CML X-XII	Patient derived xenografts	30-63	2 male, 1 female	Chronic phase CML
CML XIII-XVII	scRNA-seq $CD34^+CD38^-$ cells	17-76	2 male, 3 female	Chronic phase CML
Norm I-V	scRNA-seq $CD34^+CD38^-$ cells	19-74	2 male, 3 female	na
Norm VI-VIII	in vitro drug treatments	52-65	3 male	na

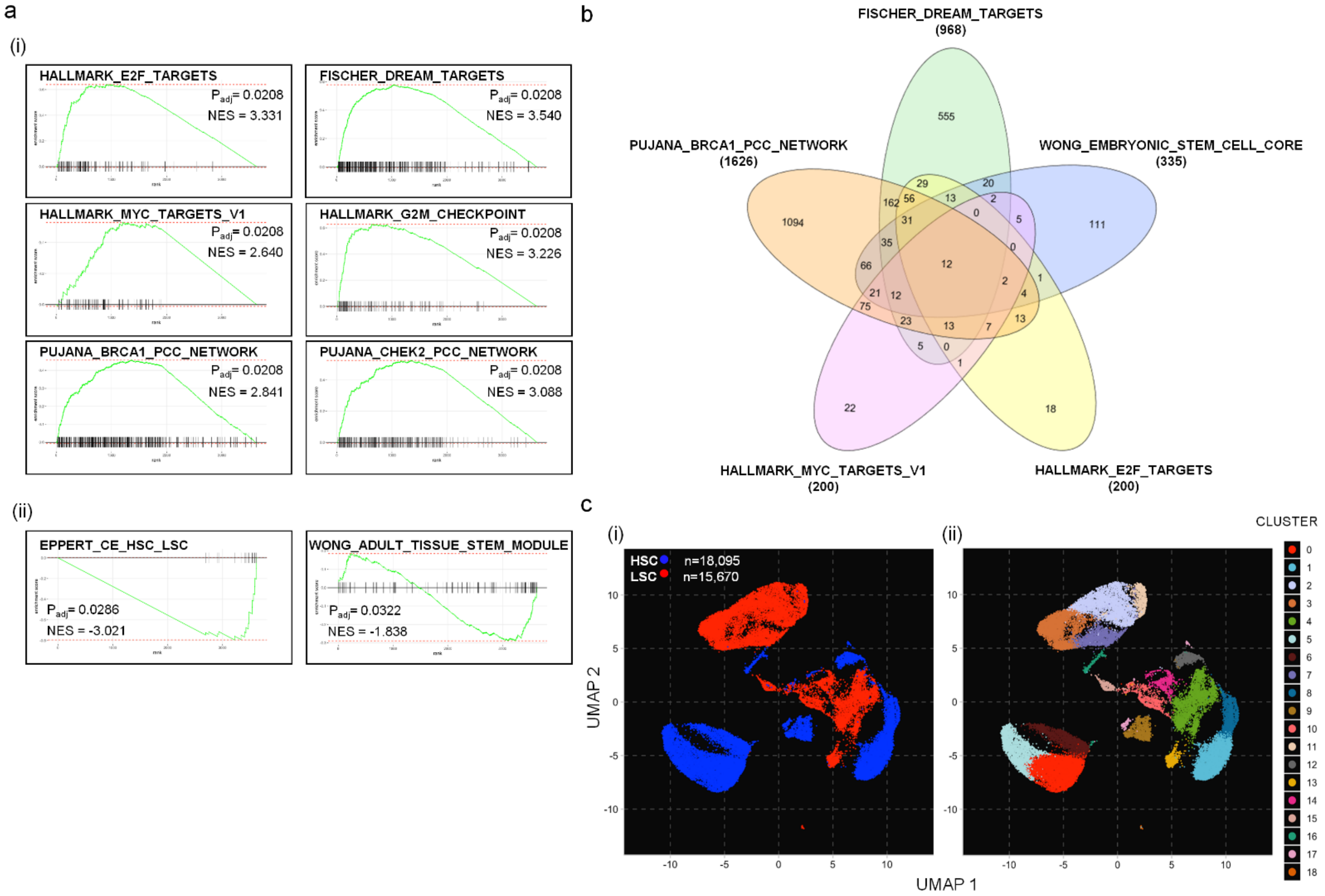

Supplementary Figure 1. A. Additional molecular signatures (MSigDB) relevant to this study identified by gene set enrichment analysis of LSC versus HSC (differentially expressed genes; E-MTAB-2581; (https://www.ebi.ac.uk/biostudies/arrayexpress/studies/E-MTAB-2581?query=E-MTAB-2581)[1]. (i) signatures found in up-regulated genes, (ii) signatures found in down-regulated genes. P_{adj} derived by Wilkinson method (EGSEA) with Benjamini-Hochberg multiple testing correction. **B.** Five-way Venn diagram showing the overlap of genes found in molecular signatures (MSigDB) relevant to this study identified by gene set enrichment analysis of LSC versus HSC. **C.** UMAP visualisation of $CD34^{+}CD38^{-}$ HSC and LSC (scRNA-seq) where a collection of stem cell genes (Supplementary Data 1) were used to derive the clusters (n=18,095 HSC and 15,670 LSC).

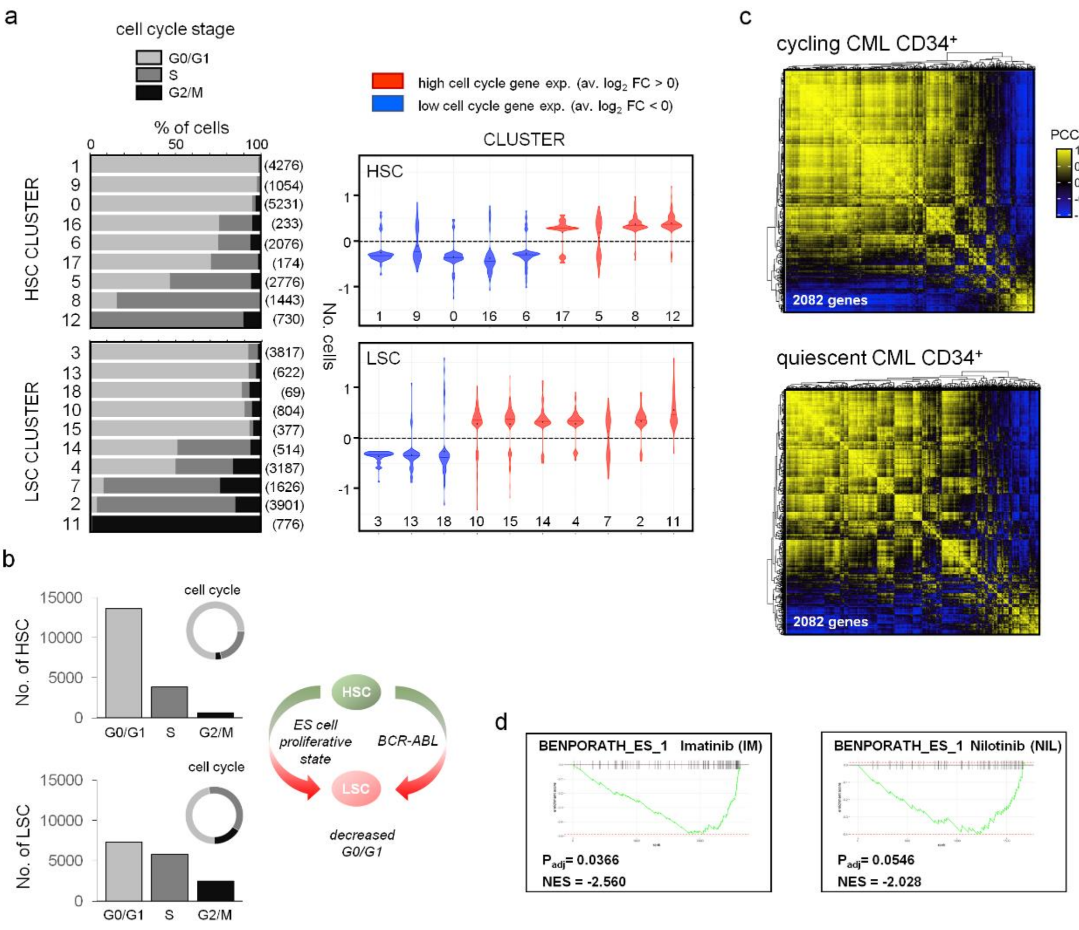

Supplementary Figure 2. A. Left: histograms showing the percentages of cells in each HSC and LSC scRNA-seq cluster (described in Fig. 1b; Supplementary Fig. 1b) with respect to phases of the cell cycle. Right: violin plots showing the mean expression of cell cycle genes (Reactome.org; depicted as mean fold change for each gene/cluster) across these clusters. **B.** Histograms and schematic diagram depicting the overall percentages of HSC or LSC within phases of the cell cycle. **C**. Heatmap depicting unsupervised clustering of Pearson correlation coefficients arising from gene-pair analysis of 2082 candidate ESC-REG genes in cycling (top) and quiescent (bottom) primary CML $CD34^+$ cells. PCC = Pearson correlation coefficient. **D.** Enrichment of ESC molecular signatures (MSigDB) in down-regulated genes upon treatment of CML LSC with TKI (imatinib or nilotinib) (7 days treatment; E-MTAB-2594; https://www.ebi.ac.uk/biostudies/arrayexpress/studies/E-MTAB-2594?query=E-MTAB-2594)[2]. P_{adj} derived by Wilkinson method (EGSEA) with Benjamini-Hochberg multiple testing correction. Source data are provided as a Source Data file.

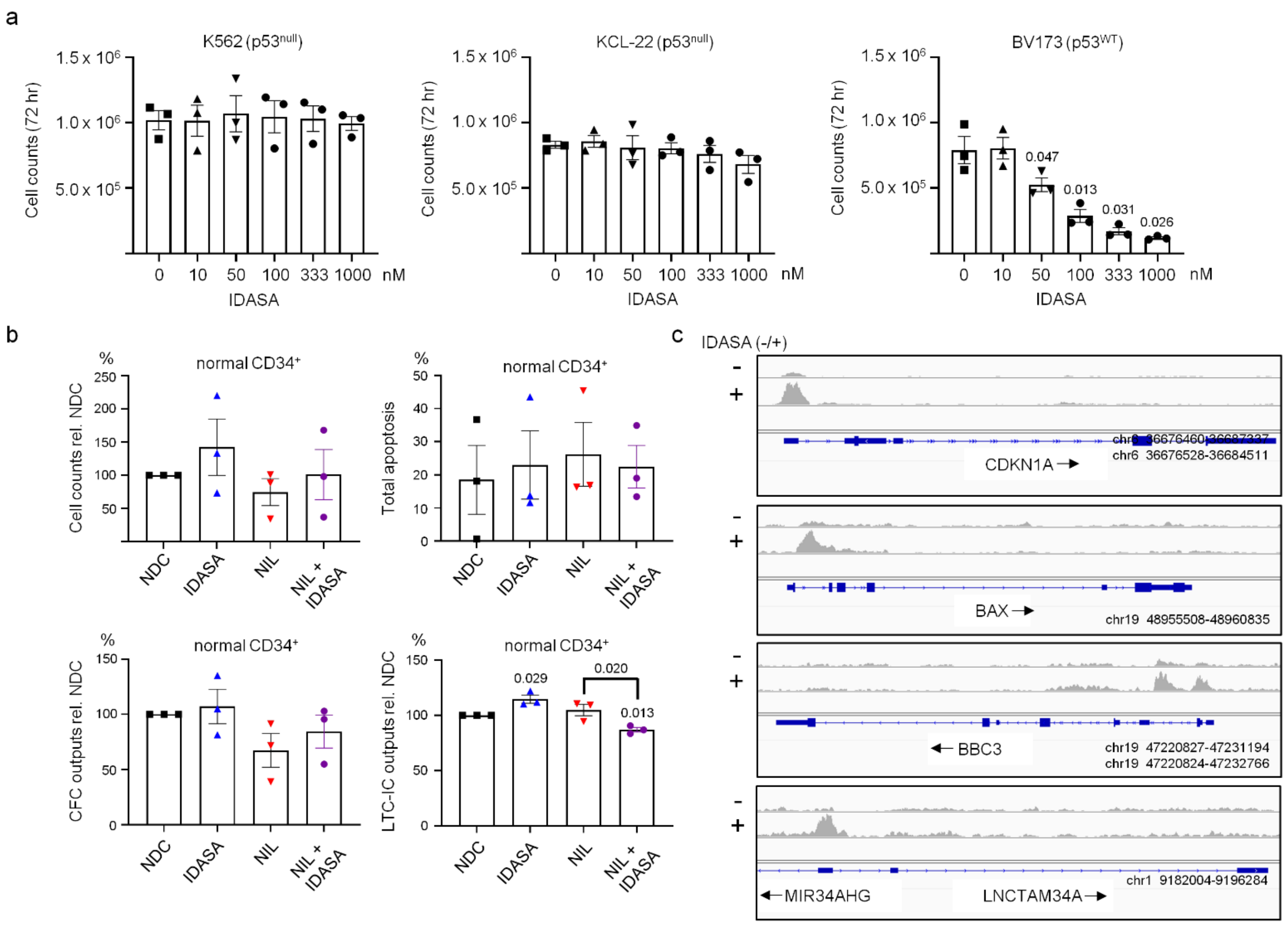

Supplementary Figure 3. A. Effect of IDASA across a range of concentrations on cell counts of the K562, KCL-22 (both p53^{null}) and BV173 (p53^{WT}) cell lines treated for 72 hrs (n=3 passages for each cell line). **B.** Effect of treatment with IDASA (100 nM) alone or in combination with NIL (3 µM) *in vitro* for 72 hours on primary normal CD34^{+} cells (n=3 independent samples). Histograms show effects on cell counts, total apoptosis (Annexin V$^{+/-}$ 7-AAD^{+} cells), clonogenic potential (CFC outputs) and on primitive cells (LTC-IC output). **C.** Visualisation (UCSC genome browser) of p53 ChIP-seq read densities at known p53 targets before (-) and after (+) treatment of BV173 cells with 300 nM idasanutlin for 6 hrs. Gene structures, genome co-ordinates and direction of transcription (arrowheads) are shown for each of the p53 targets. P values were determined using: the two-sided paired Student’s t-test (panel A), one-sided paired Student’s t-test (panel B). Error bars are SEM (panels A,B). Source data are provided as a Source Data file.

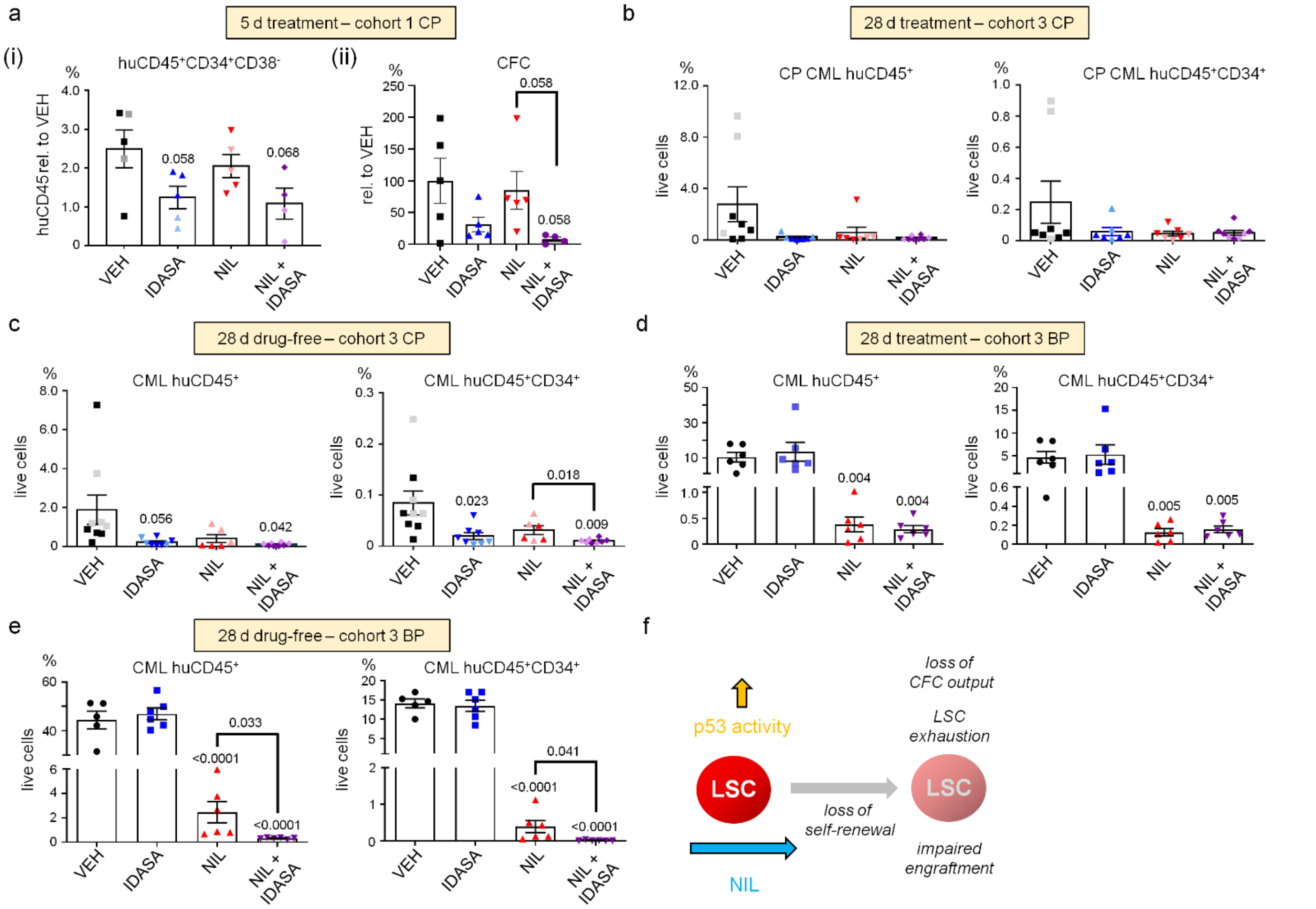

Supplementary Figure 4. Effects of treatment with nilotinib (NIL) alone or in combination with idasanutlin (IDASA) on CML cells engrafted in the BM of immunocompromised mice. See Fig. 4a for experimental design. **A.** Day 5 (cohort 1): (i) Histogram shows levels of huCD45^{+}34^{+}38^{-} LSC in murine BM expressed as a percentage of huCD45^{+} levels in vehicle only (VEH) control mice (n=5 VEH, IDASA, NIL; n=4 NIL + IDASA). (ii) Histogram shows clonogenic potential expressed as a percentage of the CFC output of VEH per 10,000 huCD45^{+} isolated by flow cytometry (n=5 VEH, IDASA, NIL; n=4 NIL + IDASA). **B.** Day 28 treatment (cohort 3; chronic phase): histograms show levels of huCD45^{+} (left) and huCD45^{+}34^{+} (right) cells in murine BM expressed as a percentage of live cells (n=8 VEH; n=7 IDASA, NIL; n=8 NIL + IDASA). **C.** Day 28 treatment-free period (cohort 3; chronic phase): histograms show levels of huCD45^{+} (left) and huCD45^{+}34^{+} (right) cells in murine BM expressed as a percentage of live cells (n=9 VEH; n=8 IDASA; n= 6 NIL; n=8 NIL + IDASA). **D.** Day 28 treatment (cohort 3; blast phase): histograms show levels of huCD45^{+} (left) and huCD45^{+}34^{+} (right) cells in murine BM expressed as a percentage of live cells (n=6/condition). **E.** Day 28 treatment-free period (cohort 3; blast phase): histograms show levels of huCD45^{+} (left) and huCD45^{+}34^{+} (right) cells in murine BM expressed as a percentage of live cells (n=6/condition). **F.** Schematic diagram depicting the functional consequences of p53 activation in LSC on a background

of nilotinib treatment. In all cases, P values determined using two-sided unpaired Student’s t-test; error bars are SEM (in panels A-E). Source data are provided as a Source Data file.

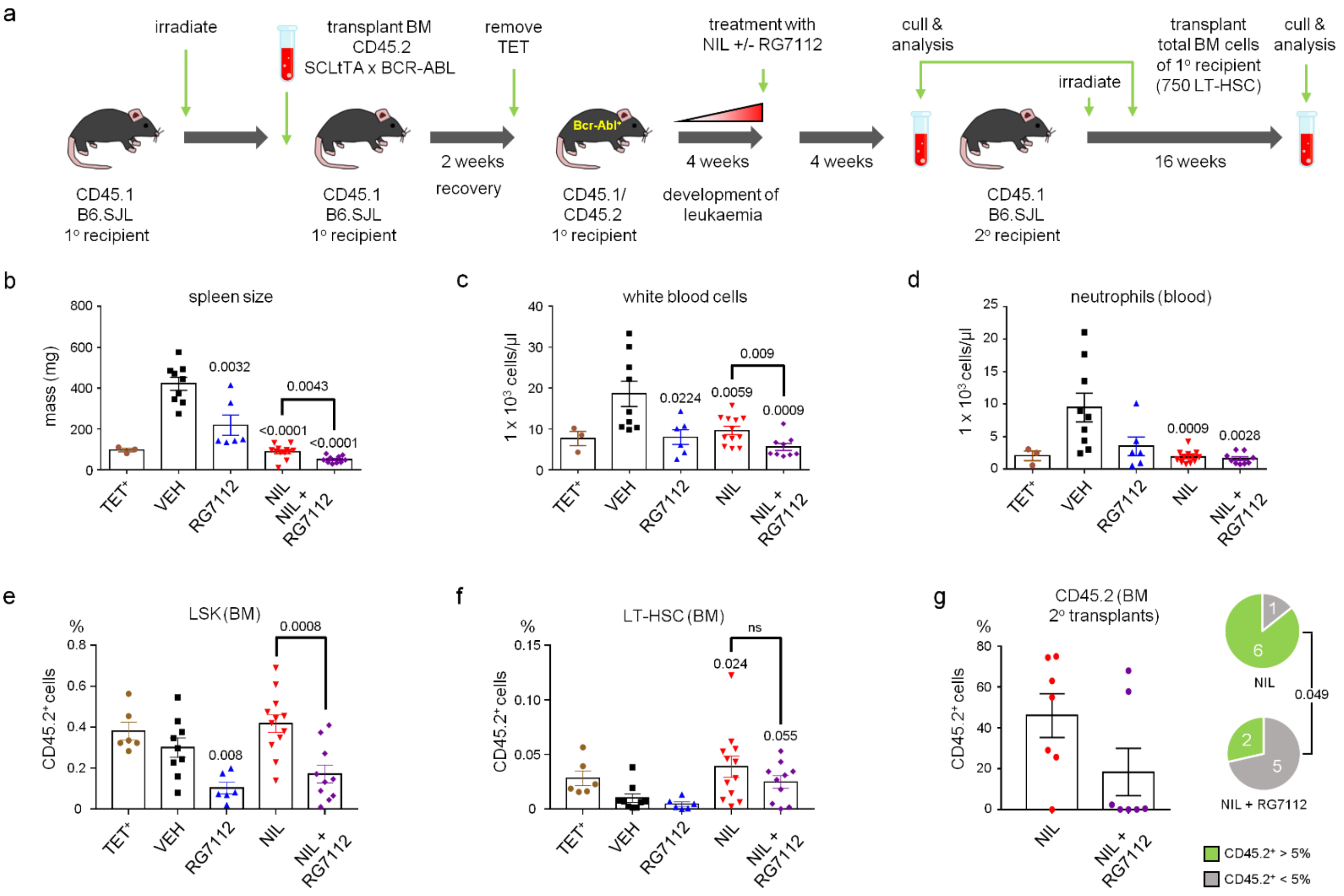

Supplementary Figure 5. A. Experiment design used to monitor the effects of treatment with nilotinib (NIL) alone or in combination with the MDM2 inhibitor RG7112 in an inducible BCR::ABL1 transgenic model. Histograms showing the effects of different treatments on: **B.** Spleen weights (n=3 TET+; n=9 VEH; n=6 RG7112; n=12 NIL; n=10 NIL + RG7112) and levels of: **C.** white blood cell (n=3 TET+; n=9 VEH; n=6 RG7112; n=12 NIL; n=9 NIL + RG7112). **D.** neutrophils (n=3 TET+; n=9 VEH; n=6 RG7112; n=12 NIL; n=9 NIL + RG7112). **E.** CD45.2$^+$ Lin$^-$Sca$^+$c-Kit$^+$ (LSK) bone marrow (BM) cells (n=6 TET+; n=9 VEH; n=6 RG7112; n=12 NIL; n=10 NIL + RG7112). **F.** CD45.2$^+$ LT-HSC BM cells (n=6 TET+; n=9 VEH; n=6 RG7112; n=12 NIL; n=10 NIL + RG7112). **G.** Engraftment of BM cells containing 750 CD45.2$^+$ LT-HSC from the NIL and NIL + RG7112 treated primary recipients into secondary recipients (n=7 NIL; n=7 NIL + RG7112). Bar graph (left panel) shows percentage donor CD45.2$^+$ cells engrafted in the BM of secondary recipients and pie charts (right panel) shows number of secondary recipients engrafted with CD45.2+ cells in the BM (using 5% CD45.2$^+$ cells as a threshold for engraftment). TET$^+$ = mice with no leukaemia induction. Each datapoint on histograms = one mouse. P values determined using the two-sided unpaired Student's t-test (panels B-F) or Chi-squared test (panel G); error bars are SEM. Source data are provided as a Source Data file.

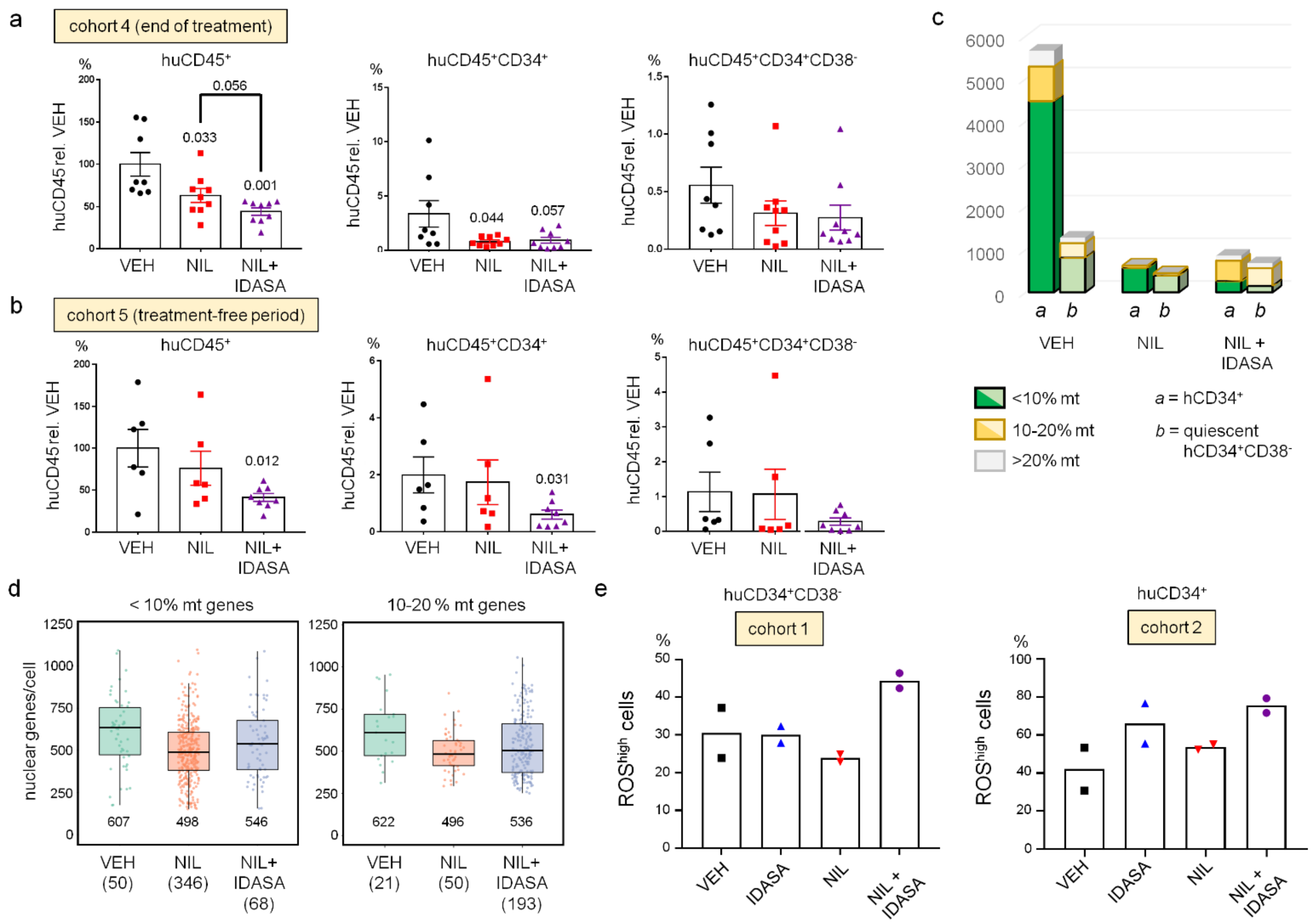

Supplementary Figure 6. See Fig. 5a for experimental design. **A.** Day 28 treatment (cohort 4): histograms show levels of huCD45^{+} (left), huCD45^{+}34^{+} (middle) and huCD45^{+}34^{+}CD38^{-} (right) cells in BM of untreated PDX mice (VEH) and PDX mice treated with nilotinib (NIL) or a combination of nilotinib plus idasanutlin (NIL + IDASA) (n=8 VEH; n=9 NIL; n=9 NIL + IDASA). **B.** Day 28 treatment-free period (cohort 5): histograms show levels of huCD45^{+} (left), huCD45^{+}34^{+} (middle) and huCD45^{+}34^{+}CD38^{-} (right) cells in BM of VEH PDX mice and PDX mice treated with NIL or a combination of NIL + IDASA (n=6 VEH; n=6 NIL; n=8 NIL + IDASA). In panels A and B, cell levels are expressed as a percentage of huCD45^{+} levels in VEH control mice. **C.** Histogram showing predicted absolute number of human CD34^{+} and quiescent ESC-REG^{low} CD34^{+}CD38^{-} cells residing in the BM of PDX mice in VEH, NIL and NIL + IDASA conditions based on scRNA-seq data shown in Fig. 5C (cohort 4). **D.** Box plots depicting the detection of nuclear genes/cell in scRNA-seq analysis of huCD34^{+} isolated from BM of PDX across the three experimental conditions (cohort 4). Cells having UMI counts for mitochondrial genes <10% of the total (left); cells having UMI counts for mitochondrial genes of 10-20% of the total (right). Mean number of nuclear genes in each condition is shown below the box plots; number of cells analysed in each condition is shown below the names of each experimental condition. Center lines show the means; box limits indicate the 25th and 75th

percentiles; whiskers extend 1.5 times the interquartile range from the 25th and 75th percentile; outliers (dots) define minima and maxima. **E.** Histograms showing the percentage of $ROS^{high}huCD45^{+}CD34^{+}CD38^{-}$ and $ROS^{high}huCD45^{+}CD34^{+}$ cells detected in PDX experiments described in Fig. 3 (cohort 1, n=2 mice/condition; cohort 2, n=2 mice/condition). P values were determined using the two-sided unpaired Student's t-test (panels A,B); error bars are SEM (panels A,B). Source data are provided as a Source Data file.

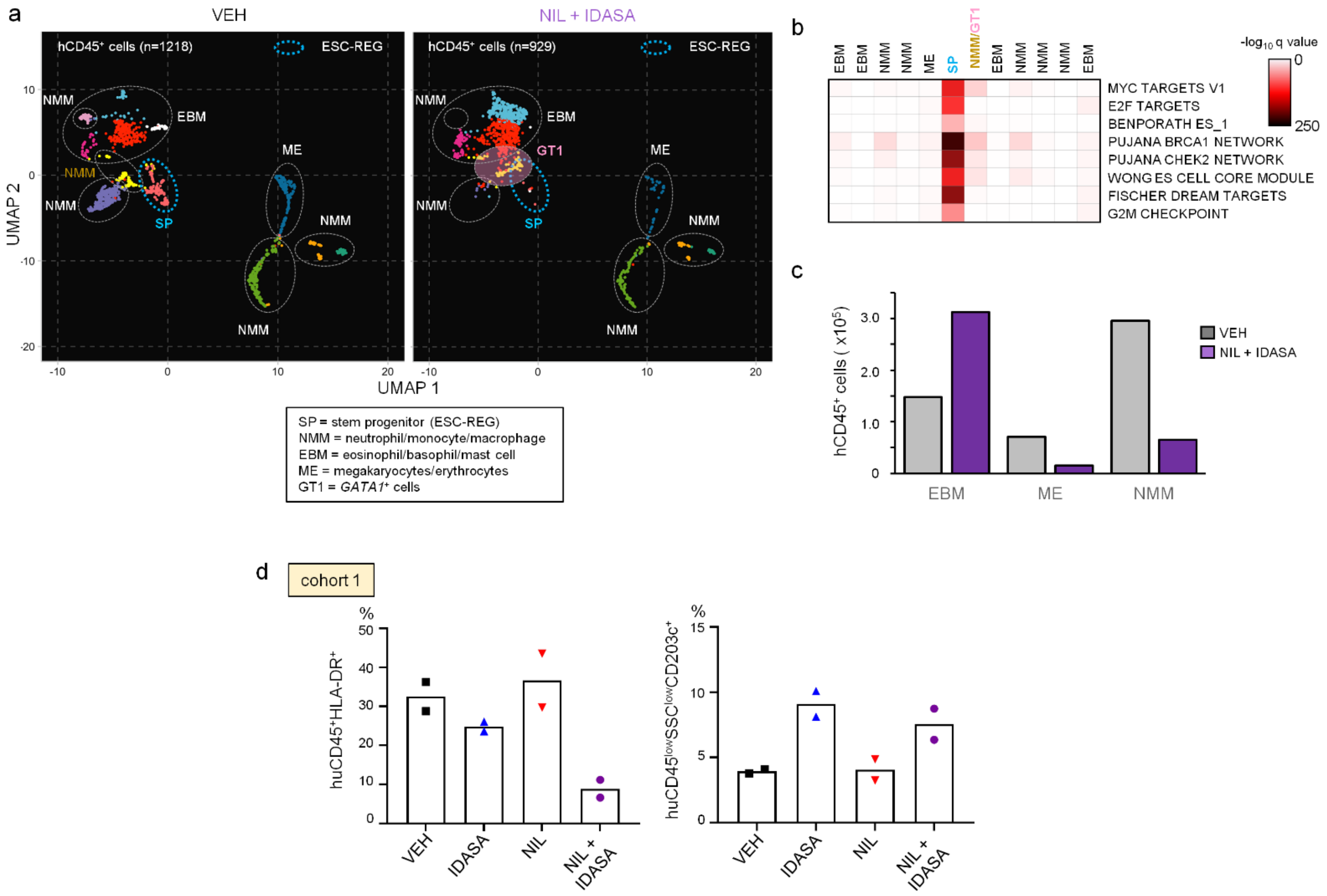

Supplementary Figure 7. A. UMAP projections depicting anchored clustering of scRNA-seq data for huCD45^{+} cells isolated from untreated (VEH) mice (n=1218 cells) and those treated with NIL + IDASA (n=929 cells) at the end of treatment (PDX; 3 mice/condition; experiment described in Fig. 6). Clusters are classified by cell type (MSigDB) enrichment scores for up-regulated marker genes, and lineages as defined elsewhere [3, 4]. The key to the lineage designations is shown below the left panel. Cluster of stem/progenitor (SP) cells enriched with marker genes found in the ESC-REG is highlighted (blue). *GATA1* (GT1) expressing cluster in the NIL + IDASA condition and described in Fig. 6 and in the text is also highlighted (pink). One cluster (NMM; highlighted in brown text) from the VEH condition, clustered with the GT1 population in the NIL + IDASA condition, although only the latter showed increased expressed of *GATA1*. **B.** Heatmap depicting enrichment values (-log$_{10}$ FDR q value derived by hypergeometric test with Benjamini-Hochberg multiple testing correction) of ESC-REG associated signatures (MSigDB) for clusters defined in panel A. Stem/progenitor (SP) cells are shown to be enriched for marker genes (up-regulated by DEG analysis) found in the ESC-REG. **C.** Histogram showing predicted absolute number of human cells residing in the BM of mice (PDX) in VEH and NIL + IDASA conditions based on scRNA-seq and lineage classifications determined in A. **D.** Histograms showing the levels of huCD45^{+}HLA-DR^{+} and huCD45^{low}SSC^{low}CD203c^{+} cells in PDX mice that have been untreated (VEH) or treated with IDASA,

NIL or NIL + IDASA. Cohort 1 refers to the experiment described in Fig. 3 (n=2 mice/condition). Source data are provided as a Source Data file.

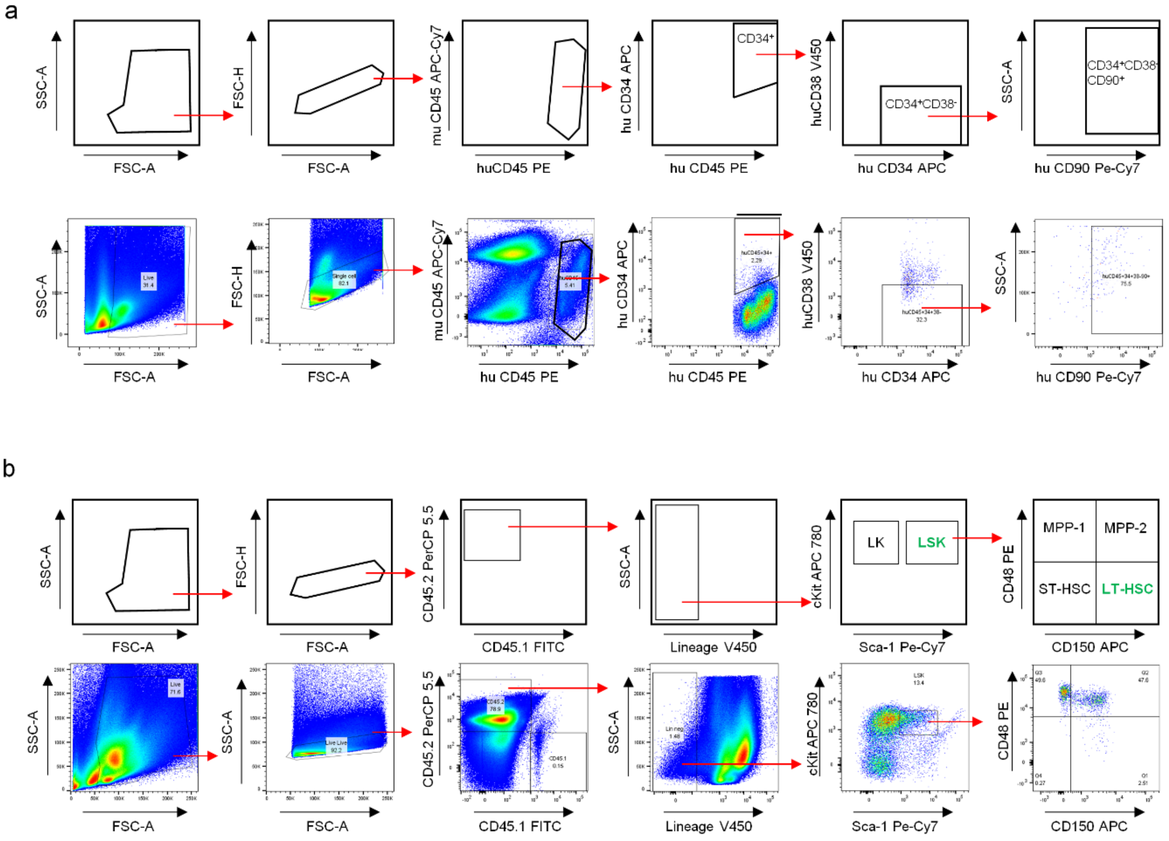

Supplementary Figure 8. FACS gating strategies used to analyse bone marrow cells in immunocompromised mice (PDX) and in the inducible BCR-ABL1 transgenic murine model of CML. **A.** Gating strategy to identify human CML cells in immunocompromised mice (PDX) **B.** Gating strategy to identify CD45.2^{+} donor cells (SCLtTA/BCR-ABL) in recipient CD45.1^{+} mice. SSC = side scatter (cell granularity). FSC = forward scatter (cell size).

REFERENCES

1. Scott MT*, et al.* Epigenetic Reprogramming Sensitizes CML Stem Cells to Combined EZH2 and Tyrosine Kinase Inhibition. *Cancer Discov* **6**, 1248-1257 (2016).

2. Pellicano F*, et al.* hsa-mir183/EGR1-mediated regulation of E2F1 is required for CML stem/progenitor cell survival. *Blood* **131**, 1532-1544 (2018).

3. Drissen R*, et al.* Distinct myeloid progenitor-differentiation pathways identified through single-cell RNA sequencing. *Nat Immunol* **17**, 666-676 (2016).

4. Hay SB, Ferchen K, Chetal K, Grimes HL, Salomonis N. The Human Cell Atlas bone marrow single-cell interactive web portal. *Exp Hematol* **68**, 51-61 (2018).
