# Supplementary material for: Activating p53 abolishes self-renewal of quiescent leukaemic stem cells in residual CML disease
Source: Nat Commun. 2024 Jan 22;15:651. doi: 10.1038/s41467-024-44771-9 (PMC10800356; doi:10.1038/s41467-024-44771-9)
Supplement: Supplementary file 2 — Reporting Summary [file 41467_2024_44771_MOESM2_ESM.pdf]

nature portfolio

Corresponding author(s): David Vetrie

Last updated by author(s): Dec 8, 2023

Reporting Summary

Nature Portfolio wishes to improve the reproducibility of the work that we publish. This form provides structure for consistency and transparency in reporting. For further information on Nature Portfolio policies, see our Editorial Policies and the Editorial Policy Checklist.

Statistics

For all statistical analyses, confirm that the following items are present in the figure legend, table legend, main text, or Methods section.

n/a	Confirmed	
☐	☒	The exact sample size (*n*) for each experimental group/condition, given as a discrete number and unit of measurement
☐	☒	A statement on whether measurements were taken from distinct samples or whether the same sample was measured repeatedly
☐	☒	The statistical test(s) used AND whether they are one- or two-sided<br>*Only common tests should be described solely by name; describe more complex techniques in the Methods section.*
☒	☐	A description of all covariates tested
☐	☒	A description of any assumptions or corrections, such as tests of normality and adjustment for multiple comparisons
☐	☒	A full description of the statistical parameters including central tendency (e.g. means) or other basic estimates (e.g. regression coefficient) AND variation (e.g. standard deviation) or associated estimates of uncertainty (e.g. confidence intervals)
☐	☒	For null hypothesis testing, the test statistic (e.g. *F*, *t*, *r*) with confidence intervals, effect sizes, degrees of freedom and *P* value noted<br>*Give P values as exact values whenever suitable.*
☒	☐	For Bayesian analysis, information on the choice of priors and Markov chain Monte Carlo settings
☐	☒	For hierarchical and complex designs, identification of the appropriate level for tests and full reporting of outcomes
☐	☒	Estimates of effect sizes (e.g. Cohen's *d*, Pearson's *r*), indicating how they were calculated

Our web collection on statistics for biologists contains articles on many of the points above.

Software and code

Policy information about availability of computer code

Data collection	Flow cytometry data was collected using a FACSVerse (BD Biosciences), FACS sorting was done using a FACSAria Fusion cell sorter (BD Biosciences). RNA libraries were then prepared using the TruSeq RNA Library Prep Kit v2 (Illumina) with single indexing following the manufacturers' instructions. ChIP-sequencing libraries were generated using the Nextera XT DNA Library Preparation Kit (Illumina) with dual indexing (Nextera XT Index Kit, Illumina) following manufacturers' instructions. For single cell RNAseq, Single cell capture and cDNA libraries were prepared using the Chromium™ Single Cell 3' and 5' gene expression library and gel bead kits v2 and v1 respectively and the Chromium™ Single Cell A Chip Kit (10X Genomics) according to the manufacturers' instructions before being processed on a Chromium Controller (10X Genomics). All sequencing was carried out using paired end sequencing on a HiSeq4000 or NovaSeq6000 (Illumina).
Data analysis	Flow Cytometry data was analysed using Flowjo version 10.8<br><br>Analysis of scRNA-sequencing data. Single cell RNA-seq data were pre-processed using the 10X Genomics Cell Ranger (3.1.0) 52 suite of analysis pipelines. Demultiplexing of raw base call (BCL) files produced by the Illumina Hiseq4000 platform and conversion to FASTQ file formats was performed using the cellranger mkfastq pipeline. Alignment of sequencing reads (FASTQ) to the GRCh38 (GENCODE v32/Ensembl 98) human genome assembly was performed using the cellranger count pipeline. Seurat (3.2.3) 53 in R (3.6.3) was used to perform QC and all further analysis and exploration of our scRNA-seq datasets. Single cell transcriptomes that had fewer than 500 genes and greater than 10% mitochondrial gene counts in CD34+CD38- cells isolated from peripheral blood samples were excluded from further analysis. Single human cells isolated from murine BM (PDX) that had fewer than 200 genes and greater than 20% mitochondrial gene counts were excluded from further analysis. All scRNA-seq datasets were normalised using "LogNormalize" with scale.factor = 10000 and "RunPCA" was used for dimensionality reduction on the scaled scRNA-seq data. We used stem cell signatures 14,25,26 (Supplemental Table S1) as PCA features for dimensionality reduction of scRNA-seq data from CML and normal CD34+CD38- cells isolated from peripheral blood. Alternatively, we used the top 2000 most highly variable genes for dimensionality reduction of CD45+ or CD45+CD34+ human cells isolated from murine BM (PDX).

The first 30 principal components from PCA results were selected to construct a K-nearest neighbour (KNN) graph by "FindNeighbors", which were clustered by Louvain algorithm from "FindClusters" thereafter. We used umap from "RunUMAP" to visualise the clustered cells. "FindMarkers" was performed to find the DEG (i.e., marker genes) of a single cluster compared with all other clusters. Cell cycle phase scores were calculated by "CellCycleScoring" based on sets of curated genes known to be expressed at different stages of the cell cycle 27. Individual cells were assigned to different stages of the cell cycle (G0/G1, S or G2/M based on their relative expression levels of curated cell cycle genes.

ESC regulome construction:To identify and construct an ESC regulome for CML cycling and quiescent LSC (CD34+CD38- cells from peripheral blood), we identified candidate regulome genes as the intersection of gene sets from relevant MSigDB 54 signatures (shown in Figure 1C) with those that were the union of differentially expressed genes of CML versus normal CD34+CD38- cells (E-MTAB-2581; ref 1) and differentially expressed between quiescent and cycling CML LSC (using clusters shown in Figure 1D). We identified 2082 genes that satisfied these criteria and for these genes we extracted normalised RMA values for cycling and quiescent CML stem and progenitor cells (E-MTAB-2508; ref 2) and performed gene-pair Pearson correlation analysis. We defined gene membership of the CML ESC regulome in cycling or quiescent CML LSC as those genes showing a mean Pearson correlation coefficient of 0.6 or greater across all the gene-pairs tested. For these highly correlated genes, we examined enrichment of adult or embryonic stem cell signatures using StemChecker 55 and projected binary (bound/unbound) TF binding profiles from mouse ESC 17. In turn, we visualised these TF-target interactions in the context of the CML ESC regulome using visNetwork (2.0.8) in R. We performed expression analysis of the CML ESC regulome genes within each stage of the cell cycle by identifying 500 exemplar cells for each cell cycle stage (G1, S, G2M, G0) using the approach outlined above 27 and then by computing cell-to-cell Pearson correlations for those cells assigned to each cell cycle stage.

Bulk-cell RNA-seq and ChIP-seq analysis: Raw sequencing data was pre-processed using the University of Glasgow Galaxy server (https://www.polyomics.gla.ac.uk/galaxy.html) for which quality control was performed by FastQC (0.72) and the reads were trimmed by Trim Galore! (0.4.3.1). For p53 ChIP-sequencing, the trimmed reads were aligned to human reference genome hg38 using Bowtie2 (2.3.4.2). Narrow peaks were called by MACS2 (2.2.7.1) with q value 0.05. Gene annotation of p53 ChIP-seq peaks employed ChIPseeker (1.28.3) (in R 3.6.3) with the annotation database TxDb.Hsapiens.UCSC.hg38.knownGene. For bulk-RNA-sequencing, the trimmed reads were aligned to human reference genome hg38 using HISAT2 (2.1.0) and assigned by the feature counting tool featureCount (1.6.0.3). DEG were obtained by DESeq2 (1.32.0) in R. Enrichment of molecular signatures in DEG identified from bulk- and single-cell RNA-seq and other transcriptomics datasets described in the Key Resources Table utilised MSigDB 54 (v7.4.1) and enrichment was calculated using enricher from R package clusterProfiler(4.0.5) with minGSSize=1, and maxGSSize=NA which means no gene set size restriction. Additionally, we used R package EGSEA (1.20.0) for the signature enrichment analysis for bulk RNA-seq data.

Statistical analysis of in vitro and in vivo phenotypic data. No datasets were excluded from the analysis and investigators were not blinded to the experimental conditions (for all in vitro assays). Statistical analyses of differences in cell expansion, CFC counts, cell cycle status and apoptosis between experimental conditions (as described above) were performed using the one-sided or two-sided paired Student's t-test as appropriate. Statistical analysis of differences in human (PDX) or murine (DTG model) cell populations between experimental conditions (as described above) were performed using the two-sided unpaired Student's t-test.

References:
1. Scott MT, et al. Epigenetic Reprogramming Sensitizes CML Stem Cells to Combined EZH2 and Tyrosine Kinase Inhibition. Cancer Discov 6, 1248-1257 (2016).
2. Graham SM, Vass JK, Holyoake TL, Graham GJ. Transcriptional analysis of quiescent and proliferating CD34+ human hemopoietic cells from normal and chronic myeloid leukemia sources. Stem Cells 25, 3111-3120 (2007).

For manuscripts utilizing custom algorithms or software that are central to the research but not yet described in published literature, software must be made available to editors and reviewers. We strongly encourage code deposition in a community repository (e.g. GitHub). See the Nature Portfolio guidelines for submitting code & software for further information.

Data

Policy information about availability of data

All manuscripts must include a data availability statement. This statement should provide the following information, where applicable:
- Accession codes, unique identifiers, or web links for publicly available datasets
- A description of any restrictions on data availability
- For clinical datasets or third party data, please ensure that the statement adheres to our policy

The expression profiling RNA-seq data generated in this study have been deposited in the Gene Expression Omnibus (GEO) database and is freely available under accession codes GSE218183 (https://www.ncbi.nlm.nih.gov/geo/query/acc.cgi?acc=GSE218183), GSE218184 (https://www.ncbi.nlm.nih.gov/geo/query/acc.cgi?acc=GSE218184), GSE218185 https://www.ncbi.nlm.nih.gov/geo/query/acc.cgi?acc=GSE218185), and GSE242036 (https://www.ncbi.nlm.nih.gov/geo/query/acc.cgi?acc=GSE242036). The ChIP-seq dataset generated in this study has been deposited in the GEO database and is freely available under accession code GSE218182 (https://www.ncbi.nlm.nih.gov/geo/query/acc.cgi?acc=GSE218182).The publicly available datasets used in this study are available in the GEO database under accession code GSE76312 (https://www.ncbi.nlm.nih.gov/geo/query/acc.cgi?acc=GSE76312) (ref 1) and in the EMBL-EBI database under accessions E-MTAB-2508 (https://www.ebi.ac.uk/biostudies/arrayexpress/studies/E-MTAB-2508) (ref 2), E-MTAB-2581 (https://www.ebi.ac.uk/biostudies/arrayexpress/studies/E-MTAB-2581?query=E-MTAB-2581) (ref 3) and E-MTAB-2594 (https://www.ebi.ac.uk/biostudies/arrayexpress/studies/E-MTAB-2594?query=E-MTAB-2594) (ref 4). Additional information concerning human samples can be obtained from the corresponding author. The remaining data are available within the Article, Supplementary Information or Source Data file.

References:
1. Giustacchini A, et al. Single-cell transcriptomics uncovers distinct molecular signatures of stem cells in chronic myeloid leukemia. Nat Med 23, 692-702 (2017).

2. Graham SM, Vass JK, Holyoake TL, Graham GJ. Transcriptional analysis of quiescent and proliferating CD34+ human hemopoietic cells from normal and chronic myeloid leukemia sources. Stem Cells 25, 3111-3120 (2007).

3. Scott MT, et al. Epigenetic Reprogramming Sensitizes CML Stem Cells to Combined EZH2 and Tyrosine Kinase Inhibition. Cancer Discov 6, 1248-1257 (2016).

4. Pellicano F, et al. hsa-mir183/EGR1-mediated regulation of E2F1 is required for CML stem/progenitor cell survival. Blood 131, 1532-1544 (2018).

Research involving human participants, their data, or biological material

Policy information about studies with human participants or human data. See also policy information about sex, gender (identity/presentation), and sexual orientation and race, ethnicity and racism.

Reporting on sex and gender	Samples used were from adults no prior knowledge of sex and gender
Reporting on race, ethnicity, or other socially relevant groupings	Samples were from adults with no prior knowledge of race or ethnicity
Population characteristics	Samples were from newly diagnosed CML patients in chronic phase prior to TKI therapy or from normal controls. Age range of patients/individuals who provided samples for this study was between 17-76 years of age. Samples from 12 males and 13 females were used in this study (data obtained retrospectively).
Recruitment	CML samples were chosen randomly for the experiments described without prior knowledge of age, sex, gender, clinical outcome or response to TKI. Normal control primary samples were obtained by mobilisation with granulocyte-colony stimulating factor (G-CSF) from individuals with no evidence of BM disease. Samples are provided by CML patients at time of diagnosis with ethical consent for the use of these samples in medical research. No compensation was provided to the patients for sample provision. Samples used in the stdy reflect the age spectrum of patients with CML, with no obvious biases likely to impact the results.
Ethics oversight	Permission to use primary samples from CML patients and normal controls for research purposes was with written informed consent in all cases (West of Scotland Research Ethics Committee 4; REC Ref 10/S0704/2 15/WS/0077, 20/WS/0066).

Note that full information on the approval of the study protocol must also be provided in the manuscript.

Field-specific reporting

Please select the one below that is the best fit for your research. If you are not sure, read the appropriate sections before making your selection.

☒ Life sciences ☐ Behavioural & social sciences ☐ Ecological, evolutionary & environmental sciences

For a reference copy of the document with all sections, see nature.com/documents/nr-reporting-summary-flat.pdf

Life sciences study design

All studies must disclose on these points even when the disclosure is negative.

Sample size	For in vivo experiments, sample size was pre-determined by performing power calculations to determine the minimum cohort sizes required for each experiment. For our in vivo studies, based on our experience, we estimate effect sizes of 50% difference between any two conditions across all our mouse model experiments/assays are sufficient to reflect real differences in leukaemia cell biology. Our cohort/condition sizes are calculated to detect these differences at a significance level of $p = 0.05$ with a standard deviation of 25% in the data for each condition, with a power of 0.8. In all instances, these considerations resulted in a minimum sample size of 4 mice per experimental condition for each experiment. Numbers of mice for each experiment are either detailed in the text/figure legend or indicated on the graph by individual points. For in vitro experiments, sample size was not pre-determined. n=3 individual samples were used for all in vitro experiments as this is the minimum required for statistical analysis.
Data exclusions	No relevant data was excluded from the analysis
Replication	For PDX work, only age-matched (8-12 week) female mice will used as recipients, as male mice are more likely to fail to engraft human CML cells and would not contribute data to the study (3Rs; refinement). For the transgenic CML model, aged-matched (8-12 week old) male and female recipients were used. For mouse experiments where cohorts of animals greater than 16 were reported, the final results reported were aggregated from 2-3 independent experiments performed 6-12 months apart where possible. All cell line and primary sample in vitro experiments were carried out in triplicate; each replicate experiment was performed independently 1-3 months apart. All attempts at replication were successful. All experiments were performed using established standard operating procedures.
Randomization	For each in vivo experiment mice were assigned randomly to each experimental arm based on engraftment levels (sampled in the blood prior to drug treatments) and sex (for transgenic model) and age-matched. For in vitro experiments, samples were used without prior knowledge to sex, age or race or clinical history with respect to treatment and all samples was subjected to the same experimental conditions (i.e., drug treatments). For in vitro experiments involving cell lines, three cryopreserved passages of cells were randomly chosen from our cell bank to ensure we performed each experiment on n = 3 independent passages.
Blinding	Investigators were not blinded to the experimental conditions. For in vivo experiments, researchers were required to be aware of the experimental arm each mouse was assigned to for monitoring purposes to comply with Home Office legislation. FACS analysis in each experiment used the same gating strategy for every mouse/ patient sample thus reducing the likelihood of bias introduced by the experimentor. For in vitro assays, the prinicipal researcher carrying out the experiment was not blinded to experimental conditions, but all CFC/LTC-IC outputs and cell counts were independently determined by another researcher who was blinded to the conditions.

Reporting for specific materials, systems and methods

We require information from authors about some types of materials, experimental systems and methods used in many studies. Here, indicate whether each material, system or method listed is relevant to your study. If you are not sure if a list item applies to your research, read the appropriate section before selecting a response.

Materials & experimental systems

n/a	Involved in the study
☐	☒ Antibodies
☐	☒ Eukaryotic cell lines
☒	☐ Palaeontology and archaeology
☐	☒ Animals and other organisms
☒	☐ Clinical data
☒	☐ Dual use research of concern
☒	☐ Plants

Methods

n/a	Involved in the study
☐	☒ ChIP-seq
☐	☒ Flow cytometry
☒	☐ MRI-based neuroimaging

Antibodies

Antibodies used

PE mouse anti-human CD45: BD Bioscience; Cat no. 555483; Clone H130; Lot: 1075455; 5 in 50 dilution
APC mouse anti-human CD34: BD Bioscience; Cat no. 555824; Clone 581; Lot: 9224663; 1 in 10 dilution
APC-Cy7 rat anti-mouse CD45: BD Bioscience; Cat. no. 557659; Clone 30-F11; Lot: 2201748; 1 in 50 dilution
V450 mouse anti-human CD38; BD Bioscience; Cat. no. 561378; Clone HIT2; Lot: 2259756; 3 in 50 dilution
PE-Cy7 mouse anti-human CD38; BD Bioscience; Cat. no. 560677; Clone HIT2; Lot: 2129025; 3 in 50 dilution
PE-Cy7 mouse anti-human CD90; BD Bioscience; Cat. no. 561558; Clone 5E10; Lot: 2312443; 1 in 50 dilution
Rat anti-mouse CD16/CD32; BD Bioscience; Cat. no. 553142; Clone 2.4G2; Lot: 8179557; 1 in 50 dilution
FITC anti-mouse CD45.1; Biolegend; Cat. no. 110706; Clone A20; Lot: B249479; 1 in 100 dilution
APC anti-mouse Ly-6G/Ly-6C (Gr-1); Biolegend; Cat. no. 108412; Clone RB6-8C5; Lot: B262855; 1 in 100 dilution
PE/Cy7 anti-mouse/human CD11b (Mac-1); Biolegend; Cat. no. 101216; Clone M1/70; Lot: B249268; 1 in 100 dilution
PE-Cy7 rat anti-mouse Ly-6A/E (Sca-1); BD Bioscience; Cat. no. 561021; Clone D7; Lot: 8341932; 1 in 100 dilution
APC anti-mouse CD150 (SLAM); Biolegend; Cat. no. 115910; Clone TC15-12F12.2; Lot: B283740; 1 in 50 dilution
PE anti-mouse CD48; Biolegend; Cat. no. 103406; Clone HM48-1; Lot: B281646; 3 in 50 dilution
PerCP/Cy5.5 anti-mouse CD45.2; Biolegend; Cat. no. 109828; Clone 104; Lot: B266798; 3 in 50 dilution
APC 780 anti-mouse CD117 (c-kit); eBiosciences; Cat. No. 47-1171-82; Clone 2B8; Lot: 2577317; 1 in 50 dilution
β-actin rabbit mAb; Cell Signalling Technology; Cat. no. 4970S; Clone 13E5; Lot 19; 1 in 1000 dilution
recombinant anti-c-Myc; Abcam; Cat. no. ab32072; Clone Y69; Lot: GR3377350-7; 1 in 1000 dilution
p53 (DO-1); Santa-Cruz Biotechnology; Cat. no. sc-126; Clone DO-1; Lot: D0412; 1 in 1000 dilution
anti-GAPDH; Abcam; Cat. no. 181602; Clone EPR16891; Lot: GR3316865-19; 1 in 5000 dilution
PE/Cy7 anti-human CD203c (E-NPP3); Biolegend; Cat. no. 324618; Clone NP4D6; Lot: B294402; 1 in 50 dilution
APC annexin V; BD Bioscience; Cat. no. 550475; Lot: 3104242; 4 in 100 dilution
APC anti-human HLA-DR; Biolegend; Cat. no. 307610; Clone L243; Lot: B372977, 1 in 50 dilution
Rabbit IgG HRP-linked antibody; Cell Signalling Technology; Cat. no. 7074; Lot:33; 1 in 5000 dilution

Validation

All antibodies have been validated by the manufacturer (BD Bioscience, Biolegend, eBioscience, Cell Signaling Technology, Santa-Cruz Biotechnology). Validation certificates can be found on the manufacturer's websites as follows:

BD Bioscience 555483 https://www.bdbiosciences.com/en-gb/products/reagents/flow-cytometry-reagents/research-reagents/single-color-antibodies-ruo/pe-mouse-anti-human-cd45.555483

BD Bioscience 555824 https://www.bdbiosciences.com/en-gb/products/reagents/flow-cytometry-reagents/research-reagents/single-color-antibodies-ruo/apc-mouse-anti-human-cd34.555824

BD Bioscience 557659 https://www.bdbiosciences.com/en-gb/products/reagents/flow-cytometry-reagents/research-reagents/single-color-antibodies-ruo/apc-cy-7-rat-anti-mouse-cd45.557659

BD Bioscience 561378 https://www.bdbiosciences.com/en-gb/products/reagents/flow-cytometry-reagents/research-reagents/single-color-antibodies-ruo/v450-mouse-anti-human-cd38.561378

BD Bioscience 560677 https://www.bdbiosciences.com/en-gb/products/reagents/flow-cytometry-reagents/research-reagents/single-color-antibodies-ruo/pe-cy-7-mouse-anti-human-cd38.560677

BD Bioscience 561558 https://www.bdbiosciences.com/en-gb/products/reagents/flow-cytometry-reagents/research-reagents/single-color-antibodies-ruo/pe-cy-7-mouse-anti-human-cd90.561558

BD Bioscience 553142 https://www.bdbiosciences.com/en-gb/products/reagents/flow-cytometry-reagents/research-reagents/single-color-antibodies-ruo/purified-rat-anti-mouse-cd16-cd32-mouse-bd-fc-block.553142

Biolegend 110706 https://www.biolegend.com/en-gb/products/fitc-anti-mouse-cd45-1-antibody-198

Biolegend 108412 https://www.biolegend.com/en-gb/products/apc-anti-mouse-ly-6g-ly-6c-gr-1-antibody-456

Biolegend 101216 https://www.biolegend.com/en-gb/products/pe-cyanine7-anti-mouse-human-cd11b-antibody-1921

BD Bioscience 561021 https://www.bdbiosciences.com/en-gb/products/reagents/flow-cytometry-reagents/research-reagents/single-color-antibodies-ruo/pe-cy-7-rat-anti-mouse-ly-6a-e.561021

Biolegend 115910 https://www.biolegend.com/en-gb/products/apc-anti-mouse-cd150-slam-antibody-2894

Biolegend 103406 https://www.biolegend.com/en-gb/products/pe-anti-mouse-cd48-antibody-293

Biolegend 109828 https://www.biolegend.com/en-gb/products/percp-cyanine5-5-anti-mouse-cd452-antibody-4271

eBiosciences 47-1171-82 https://www.thermofisher.com/antibody/product/CD117-c-Kit-Antibody-clone-2B8-Monoclonal/47-1171-82

Cell Signalling Technology 4970S https://www.cellsignal.com/products/primary-antibodies/b-actin-13e5-rabbit-mab/4970?gclid=EAIaIQobChMIoO2B8_L6ggMVHY9QBh1ilgWtEAAYASAAEgK3bvD_BwE&gclsrc=aw.ds

Abcam ab32072 https://www.abcam.com/products/primary-antibodies/c-myc-antibody-y69-chip-grade-ab32072.html

Santa-Cruz Biotechnology sc-126 https://www.scbt.com/p/p53-antibody-do-1?requestFrom=search

Abcam 181602 https://www.abcam.com/products?keywords=GAPDH+181602

Biolegend 324618 https://www.biolegend.com/en-gb/products/pe-cyanine7-anti-human-cd203c-e-npp3-antibody-8734

BD Bioscience 550475 https://www.bdbiosciences.com/en-gb/products/reagents/flow-cytometry-reagents/research-reagents/single-color-antibodies-ruo/apc-annexin-v.550475

Biolegend 307610 https://www.biolegend.com/en-gb/products/apc-anti-human-hla-dr-antibody-787

Cell Signalling Technology 7074 https://www.cellsignal.com/product/productDetail.jsp?productId=7074

Eukaryotic cell lines

Policy information about cell lines and Sex and Gender in Research

Cell line source(s)	The BV173 cell line, CML lymphoid blast crisis; obtained from DSMZ<br>The K562 cell line, CML myeloid blast crisis; obtained from DSMZ<br>The KCL-22 cell line, CML myeloid blast crisis; obtained from DSMZ
Authentication	Authenticated by Northgene Ltd, Newcastle (UK) using short-tandem repeat (STR) profiling
Mycoplasma contamination	Cells are mycoplasma negative; tested in-house on a regular basis
Commonly misidentified lines (See ICLAC register)	No commonly misidentified cell lines were used in the study

Animals and other research organisms

Policy information about studies involving animals; ARRIVE guidelines recommended for reporting animal research, and Sex and Gender in Research

Laboratory animals	Mouse: NSG (NOD.Cg-Prkdcscid Il2rgtm1Wjl/SzJ), Jackson Laboratory, Stock No: 005557 (Female 8-12 weeks at start of experiment)<br>Mouse: NRG- W41/W41 (NOD.Cg-Rag1tm1Mom KitW-41J Il2rgtm1Wjl/EavJ), Connie Eaves, Terry Fox Laboratory BC Cancer Agency (Female, 8-12 weeks at start of experiment)<br>Mouse: NRG- W41/W41 -SGM3 (NOD.Cg-Rag1tm1Mom KitW-41J Il2rgtm1Wjl Tg(CMV-IL3,CSF2,KITLG)1Eav/J), Derived in-house (Female, 8-12 weeks at start of experiment)<br>Mouse: SCL-tTA/BCR-ABL (DTG), David Vetrie Laboratory, University of Glasgow (Male and female, 8-15 weeks at start of experiment)<br>Mouse: B6.SJL-PtprcaPepcb/BoyCrCrl, Charles River Laboratory, Cat# 564 (Male and Female, 8-12 weeks at start of experiment)<br>All animals were kept under the following conditions: Light/darkness: 12/12hr, Temperature: 22+\-2, Humidity: 45-65%

Wild animals	No wild animals were used in this study
Reporting on sex	SCL-tTA/BCR-ABL (DTG) and B6.SJL-PtprcaPepcb/BoyCrCrl were male and female mice (8-12 weeks old at start of each experiment). NSG, NRG- W41/W41, NRG- W41/W41 -SGM3 were female mice (8-12 weeks old at start of the each experiment).
Field-collected samples	No field-collected samples were used in this study
Ethics oversight	For mouse experiments, all experimental protocols were approved by the local AWERB committee and national Home Office (PD6C67A47), and all methods were carried out in accordance with standard animal housing conditions under local and UK Home Office regulations

Note that full information on the approval of the study protocol must also be provided in the manuscript.

Plants

Seed stocks	No seeds were used in this study
Novel plant genotypes	No plants were used in this study
Authentication	No plants were used in this study

ChIP-seq

Data deposition

☒ Confirm that both raw and final processed data have been deposited in a public database such as GEO.

☒ Confirm that you have deposited or provided access to graph files (e.g. BED files) for the called peaks.

Data access links
May remain private before publication.

ChIP-seq datasets described in the manuscript text, figures and supplemental figures have been uploaded to GEO with the following accession number:

GSE218182 (p53 ChIP-seq in BV173 cells +/- idasanutlin; Supplemental Figure S4) ,

Direct links and tokens for reviewer access are as follows:

https://www.ncbi.nlm.nih.gov/geo/query/acc.cgi?acc=GSE218182 (token: mrspauwurxcplkv)

These data will be released to the scientific community/general public upon publication. Any further queries/requests regarding these datasets can also be made by contacting the corresponding author (david.vetrie@glasgow.ac.uk).

Files in database submission

File name	Library Name
005-036_S36_ME_R1_001.fastq.gz	BV173 - IDASA p53 ChIP
005-036_S36_ME_R2_001.fastq.gz	BV173 - IDASA p53 ChIP
annotation_005-036-S36_peaks.csv	BV173 - IDASA p53 ChIP
annotation_005-036_S36_peaks.bed	BV173 - IDASA p53 ChIP
005-037_S37_ME_R1_001.fastq.gz	BV173 + IDASA p53 ChiP
005-037_S37_ME_R2_001.fastq.gz	BV173 + IDASA p53 ChiP
annotation_005-037_S37_peaks.csv	BV173 + IDASA p53 ChiP
annotation_005-037_S37_peaks.bed	BV173 + IDASA p53 ChiP
005-039_S39_ME_R1_001.fastq.gz	BV173 - IDASA input
005-039_S39_ME_R2_001.fastq.gz	BV173 - IDASA input
005-041_S41_ME_R1_001.fastq.gz	BV173 + IDASA input
005-041_S41_ME_R2_001.fastq.gz	BV173 + IDASA input

Genome browser session
(e.g. UCSC)

https://genome.ucsc.edu/s/VetrieLab/BV173%20ChIP%20Datasets_Scott%20et%20al

Methodology

Replicates	n = 1 replicate of p53 ChIP for BV173 cells without idasanutlin; n = 1 replicate of p53 ChIP for BV173 cells with idasanutlin; n = 1 replicate of input DNA for BV173 cells without idasanutlin; n = 1 replicate of input DNA for BV173 cells with idasanutlin;
Sequencing depth	BV173 - IDASA p53 ChIP paired-end (75 bp) 39 520 109 reads (R1); 40 966 760 (R2)

BV173 + IDASA p53 ChiP	paired-end (75 bp)	39 410 306 reads (R1); 41 397 228 reads (R2)
BV173 - IDASA input	paired-end (75 bp)	40 623 738 reads (R1); 42 802 649 reads (R2)
BV173 + IDASA input	paired-end (75 bp)	41 457 489 reads (R1); 43 839 302 reads (R2)

Antibodies

p53 antibody (clone DO-1, sc-126, Santa Cruz Biotechnology, lot no. D0915)

Peak calling parameters

Narrow peaks (default settings) were called by MACS2 (2.2.7.1) with q value 0.05.

Data quality

BV173 - IDASA p53 ChIP	total peaks called (q<0.05) = 119; peaks > 5 fold enrichment = 80; total gene targets (q<0.05) = 88
BV173 + IDASA p53 ChIP	total peaks called (q<0.05) = 2161; peaks > 5 fold enrichment = 609; total gene targets (q<0.05) = 1688

Software

Raw sequencing data was pre-processed using the University of Glasgow Galaxy server (https://www.polyomics.gla.ac.uk/galaxy.html) for which quality control was performed by FastQC (0.72) and the reads were trimmed by Trim Galore! (0.4.3.1). For p53 ChIP-sequencing, the trimmed reads were aligned to human reference genome hg38 using Bowtie2 (2.3.4.2). Narrow peaks were called by MACS2 (2.2.7.1) with q value 0.05. Gene annotation of p53 ChIP-seq peaks employed ChIPseeker (1.28.3) (in R 3.6.3) with the annotation database TxDb.Hsapiens.UCSC.hg38.knownGene.

Flow Cytometry

Plots

Confirm that:

- [ ] The axis labels state the marker and fluorochrome used (e.g. CD4-FITC).
- [ ] The axis scales are clearly visible. Include numbers along axes only for bottom left plot of group (a 'group' is an analysis of identical markers).
- [ ] All plots are contour plots with outliers or pseudocolor plots.
- [x] A numerical value for number of cells or percentage (with statistics) is provided.

Methodology

Sample preparation

For in vivo experiments cell suspensions were prepared from BM harvested from the two hind legs (ilia, femurs, and tibias) of each mouse by crushing in a mortar and pestle. Cells were resuspended in 2% FBS/PBS solution, filtered and stained with the antibodies described in the methods and/or figure legends.
For in vitro experiments, cells in culture were pelleted at 1200 rpm/5 mins, media was removed and cells washed with 5 ml PBS/2%FCS. Following removal of the wash, the cells were stained with the antibodies detailed in the methods and figure legends.
Stained cells were analysed on FACSVerseTM Flow Cytometer (BD Biosciences).

Instrument

Flow cytometry data was collected using a FACSVerse (BD Biosciences)

Software

Flow cytometry data was analysed using Flowjo version 10.8

Cell population abundance

For huCD34+38- cells sorted from primary human CD34+ samples, approx 10% of the population was gated
For huCD45+ cells sorted from the BM of PDX mice, approx 1-5% of the total BM population was gated
For huCD34+ cells sorted from the BM of PDX mice, approx. 0.1-0.5% of the population was gated
In each case cell purity was approx 90-95%

Gating strategy

For all flow cytometry assays, cells were first gated by FSC/SSC to exclude debris and FSC-A/FSC-H to remove doublets. For sorting a live/dead stain was included. Single colour controls were used to identify positive and negative populations and FMO's were used where required. Full gating strategies can be supplied by corresponding author if required.

- [x] Tick this box to confirm that a figure exemplifying the gating strategy is provided in the Supplementary Information.
