# Supplementary material for: Activating p53 abolishes self-renewal of quiescent leukaemic stem cells in residual CML disease
Source: Nat Commun. 2024 Jan 22;15:651. doi: 10.1038/s41467-024-44771-9 (PMC10800356; doi:10.1038/s41467-024-44771-9)
Supplement: Supplementary file 3 — Description of Additional Supplementary Files [file 41467_2024_44771_MOESM3_ESM.pdf]

DESCRIPTION OF ADDITIONAL SUPPLEMENTARY FILES

Title: Supplementary Data 1.
Description: Curated stem cell genes relevant to this study. Worksheet 1: Stem cell genes curated from molecular signatures in MSigDB[1] or from previously published studies[2, 3, 4]. **Worksheet 2:** Sub-set of stem cell genes from worksheet 1 detected in scRNA-seq data for $CD34^{+}CD38^{-}$ HSC and LSC detailed in Supplementary Fig. 1b and in the main text.

Title: Supplementary Data 2.
Description: Genes highly correlated (mean PCC > 0.60 or less than -0.6) by mRNA expression in cycling and quiescent chronic phase CML $CD34^{+}$ cells (E-MTAB-2508; https://www.ebi.ac.uk/biostudies/arrayexpress/studies/E-MTAB-2508?query=E-MTAB-2508)[5]. Worksheet 1: Genes correlated in cycling cells. **Worksheet 2:** Genes correlated in quiescent cells. **Worksheet 3:** ESC-REG genes (human genes and murine orthologues) and their predicted transcription factor binding profiles. Transcription factor binding profiles for murine ESC (GSE20551; https://www.omicsdi.org/dataset/geo/GSE20551)[6] were obtained from a previously published study. 0 = no binding; 1 = binding.

Title: Supplementary Data 3.
Description: DEG from the ESC-REG when $CD34^{+}CD38^{-}$ LSC were treated with TKIs *in vitro*. Worksheet 1: Affymetrix probes, Ensembl gene IDs, HGNC symbols and expression changes ($\log_2$ fold changes) observed for 8 hr and 7 day treatments with imatinib or dasatinib or nilotinib (E-MTAB-2594; https://www.ebi.ac.uk/biostudies/arrayexpress/studies/E-MTAB-2594?query=E-MTAB-2594)[7] compared to the 0 hr timepoint. Each of these 465 genes was significantly differentially expressed (P_{adj} <0.05; calculated using Moderated t-test with Benjamin-Hochberg multiple testing correction) in at least 1 of the 6 conditions reported here.

Title: Supplementary Data 4.
Description: DEG when CML $CD34^{+}$ cells were treated with idasanutlin (IDASA) or nilotinib (NIL) alone or in combination *in vitro*. Worksheet 1: DEG using Ensembl gene annotation showing HGNC symbols and expression changes ($\log_2$ fold changes) observed for 24 hr and 72 hr treatments: IDASA vs no drug control (NDC), NIL versus NDC, IDASA + NIL vs NDC, and IDASA + NIL vs NIL. **Worksheet 2:** DEG using NCBI gene annotation showing HGNC symbols and expression changes ($\log_2$ fold changes) observed for 24 hr and 72 hr treatments for IDASA vs no drug control (NDC), NIL versus NDC, IDASA + NIL vs NDC, and IDASA + NIL vs NIL. Genes shown were significantly differentially expressed (P_{adj} <0.1; calculated using Wald test with Benjamini-Hochberg multiple testing correction) in at least 1 of the 4 conditions reported here.

Title: Supplementary Data 5.
Description: p53 ChIP-seq analysis for BV173 cells treated with idasanutlin for 6 hrs. Worksheet 1: ChipSeeker (https://bioconductor.org/packages/release/bioc/html/ChIPseeker.html) annotation for

p53 ChIP-seq analysis. **Worksheet 2:** All p53 targets induced in wild type p53 BV173 cells treated with idasanutlin.

Title: Supplementary Data 6.
Description: Molecular signatures observed in human ESC-REG^{low} LSC upon treatment of PDX mice with nilotinib (NIL) or nilotinib plus idasanutlin (NIL + IDASA). Worksheet 1: Oxidative stress signature identified at the end of 28 day treatment (cohort 4 described in Fig. 6 and the text). **Worksheet 2:** Erythroid signature identified at the end of 28 day treatment-free period (cohort 5 described in Fig. 6 and the text). Genes shown for each signature were significantly differentially expressed (P_{adj} <0.05; calculated using the Wilcoxon rank sum test with Bonferroni multiple testing correction) in human ESC-REG^{low} LSC isolated from the NIL + IDASA condition when compared with the NIL only condition in both cohorts.

Title: Supplementary Data 7.
Description: Worksheet 1: All reagents used in this study as mentioned in Methods.

Title: Supplementary Data 8.
Description: Worksheet 1: All antibodies used in the study (including suppliers, catalogue numbers, clone numbers, lot numbers, and dilutions used) as mentioned in Methods.

REFERENCES

1. Liberzon A, Subramanian A, Pinchback R, Thorvaldsdottir H, Tamayo P, Mesirov JP. Molecular signatures database (MSigDB) 3.0. *Bioinformatics* **27**, 1739-1740 (2011).

2. Wong DJ, Liu H, Ridky TW, Cassarino D, Segal E, Chang HY. Module map of stem cell genes guides creation of epithelial cancer stem cells. *Cell Stem Cell* **2**, 333-344 (2008).

3. Eppert K*, et al.* Stem cell gene expression programs influence clinical outcome in human leukemia. *Nat Med* **17**, 1086-1093 (2011).

4. Jaatinen T*, et al.* Global gene expression profile of human cord blood-derived CD133+ cells. *Stem Cells* **24**, 631-641 (2006).

5. Graham SM, Vass JK, Holyoake TL, Graham GJ. Transcriptional analysis of quiescent and proliferating CD34+ human hemopoietic cells from normal and chronic myeloid leukemia sources. *Stem Cells* **25**, 3111-3120 (2007).

6. Kim J, *et al.* A Myc network accounts for similarities between embryonic stem and cancer cell transcription programs. *Cell* **143**, 313-324 (2010).

7. Pellicano F, *et al.* hsa-mir183/EGR1-mediated regulation of E2F1 is required for CML stem/progenitor cell survival. *Blood* **131**, 1532-1544 (2018).
